# Data Resource Profile: The Information System for Research in Primary Care (SIDIAP)

**DOI:** 10.1093/ije/dyac068

**Published:** 2022-04-13

**Authors:** Martina Recalde, Clara Rodríguez, Edward Burn, Marc Far, Darío García, Jordi Carrere-Molina, Mencia Benítez, Anna Moleras, Andrea Pistillo, Bonaventura Bolíbar, María Aragón, Talita Duarte-Salles

**Affiliations:** Fundació Institut Universitari per a la Recerca a l'Atenció Primària de Salut Jordi Gol i Gurina (IDIAPJGol), Barcelona, Spain; Universitat Autònoma de Barcelona, Bellaterra, Spain; Fundació Institut Universitari per a la Recerca a l'Atenció Primària de Salut Jordi Gol i Gurina (IDIAPJGol), Barcelona, Spain; Fundació Institut Universitari per a la Recerca a l'Atenció Primària de Salut Jordi Gol i Gurina (IDIAPJGol), Barcelona, Spain; Centre for Statistics in Medicine, NDORMS, University of Oxford, Oxford, UK; Fundació Institut Universitari per a la Recerca a l'Atenció Primària de Salut Jordi Gol i Gurina (IDIAPJGol), Barcelona, Spain; Fundació Institut Universitari per a la Recerca a l'Atenció Primària de Salut Jordi Gol i Gurina (IDIAPJGol), Barcelona, Spain; Fundació Institut Universitari per a la Recerca a l'Atenció Primària de Salut Jordi Gol i Gurina (IDIAPJGol), Barcelona, Spain; Fundació Institut Universitari per a la Recerca a l'Atenció Primària de Salut Jordi Gol i Gurina (IDIAPJGol), Barcelona, Spain; Fundació Institut Universitari per a la Recerca a l'Atenció Primària de Salut Jordi Gol i Gurina (IDIAPJGol), Barcelona, Spain; Fundació Institut Universitari per a la Recerca a l'Atenció Primària de Salut Jordi Gol i Gurina (IDIAPJGol), Barcelona, Spain; Fundació Institut Universitari per a la Recerca a l'Atenció Primària de Salut Jordi Gol i Gurina (IDIAPJGol), Barcelona, Spain; Universitat Autònoma de Barcelona, Bellaterra, Spain; Fundació Institut Universitari per a la Recerca a l'Atenció Primària de Salut Jordi Gol i Gurina (IDIAPJGol), Barcelona, Spain; Fundació Institut Universitari per a la Recerca a l'Atenció Primària de Salut Jordi Gol i Gurina (IDIAPJGol), Barcelona, Spain

Key Features

- The Information System for Research in Primary Care (SIDIAP) is a database of population-wide primary care electronic health records that was created to provide a useful tool for healthcare research.
- SIDIAP includes data from 328 primary care centres managed by the Catalan Health Institute in Catalonia, Spain. The database contains pseudo-anonymized records for >8 million people since 2006, with 5.8 million people active in June 2021 (75% of the Catalan population). SIDIAP is representative of the general population living in Catalonia in terms of age, sex and geographic distribution.
- SIDIAP is updated on a 6-monthly basis and the median follow-up time of the population is currently 15.5 years.
- SIDIAP includes high-quality data on demographics, all-cause mortality, disease diagnoses, prescription and dispensation of drugs, laboratory tests, socio-economic indicators, vaccinations, lifestyle information, parent–child linkage and clinical parameters, among others. SIDIAP can be linked on a project-by-project basis to other data sources such as hospital discharges, mental health centres or specific disease registries.
- Researchers from public institutions can request data access if they comply with certain requirements. Further information is available online (www.sidiap.org).

## Data resource basics

### Primary care in Catalonia, Spain

Spain has a universal taxpayer-funded health system that is decentralized to its 17 autonomous communities (the country's first level of political and administrative division).^1^ Primary care is free of charge and is the main entry point for accessing public non-emergency health-related services that are delivered through primary care. Persons can be referred to secondary care if necessary. Primary care centres are composed of general practitioners (GPs), paediatricians, dentists, nurses, social workers, auxiliary nurses and administrative staff. Additionally, as part of primary care, there are a set of support services such as sexual and reproductive health or home care at the end of life.

**Figure 1:**
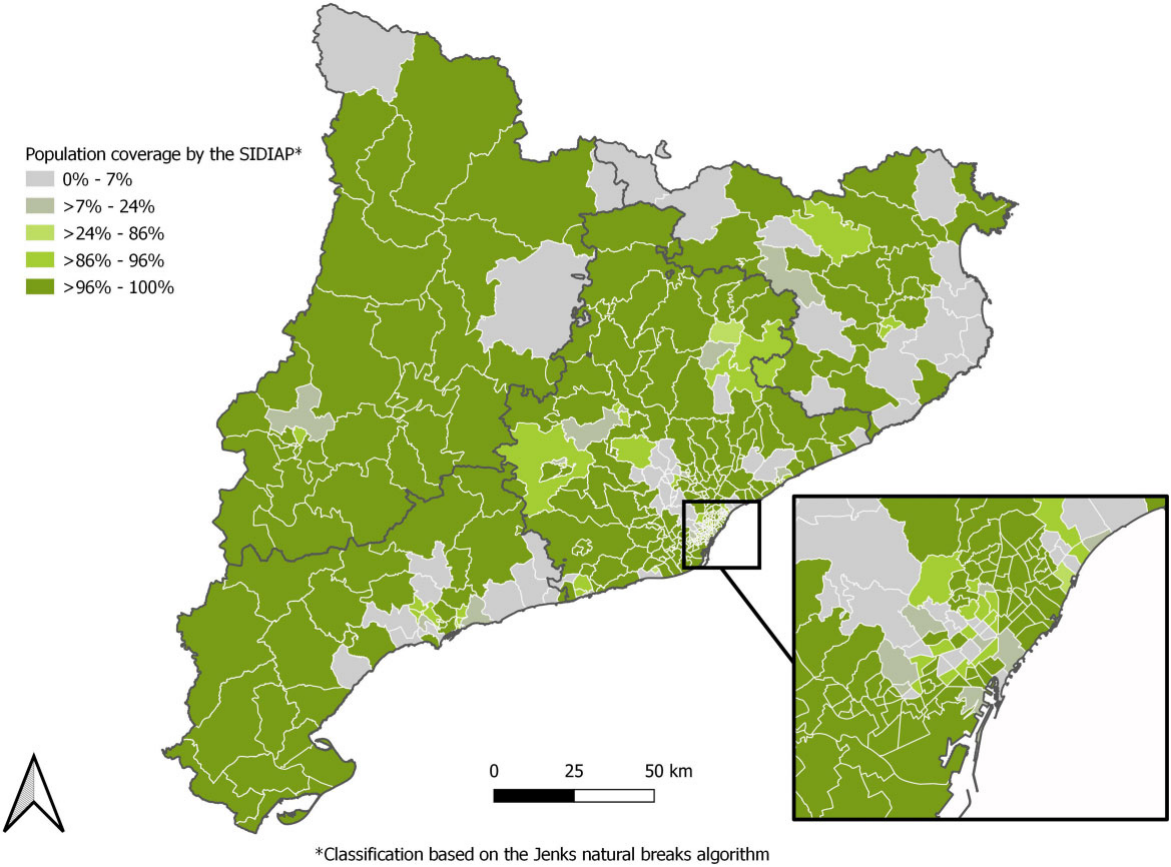
Population coverage by the Information System for Research in Primary Care database by Basic Health Area on 30 June 2021 Basic Health Areas are the territorial units by which primary healthcare services are organized in Catalonia. This delimitation is determined by the population's accessibility to health services, the efficiency of the organization of health resources and other factors (geographical, demographical, social and epidemiological).

In Catalonia, an autonomous community in north-eastern Spain, the *Institut Català de la Salut* (ICS, Catalan Health Institute) is the largest healthcare provider.^2^ As seen in Figure 1, ICS covers most of the Basic Health Areas (territorial units by which primary healthcare services are organized in Catalonia) across Catalonia. It manages 328 primary care centres covering 5.8 million people, 75% of the population living in Catalonia (the remaining 25% is distributed among other providers whose services are hired by the Department of Health) (Figure 1 and Table 1).

**Table 1:** Socio-demographic characteristics of the Information System for Research in Primary Care population, all-time and on 30 June 2021

	SIDIAP population	
	Total since 2006	Current (as of 30 June 2021)
Persons, *n*	8 036 948	5 801 280
Coverage of the population of Catalonia, %	–	74.9
Follow-up in years, median (IQR)	15.2 (6.2–15.5)	15.5 (12.7–15.5)
Sex, *n* (%)		
Female	4 029 112 (50.1)	2 940 521 (50.7)
Male	4 007 836 (49.9)	2 860 759 (49.3)
Age in years, median (IQR)	44 (27–63)	44 (25–60)
Age in years, *n* (%)		
<18	1 245 702 (15.5)	1 009 303 (17.4)
18–64	4 912 030 (61.1)	3 676 712 (63.4)
>64	1 879 216 (23.4)	1 115 265 (19.2)
Geographic region of nationality, *n* (%)		
Spain	6 476 556 (80.6)	4 867 045 (83.9)
Europe (other than Spain)	409 026 (5.1)	241 816 (4.2)
America	500 886 (6.2)	278 905 (4.8)
Asia	252 291 (3.1)	156 298 (2.7)
Africa	397 178 (4.9)	256 687 (4.4)
Oceania	1011 (0.0)	529 (0.0)
Type of residential area, *n* (%)		
Urban	6 652 818 (82.8)	5 125 779 (88.4)
Rural	433 639 (5.4)	332 827 (5.7)
Missing	950 491 (11.8)	342 674 (5.9)
Catalan regions, *n* (%)		
Barcelona	5 733 291 (71.3)	4 365 572 (75.3)
Lleida	518 810 (6.5)	390 365 (6.7)
Girona	667 816 (8.3)	512 985 (8.8)
Tarragona	706 192 (8.8)	527 997 (9.1)
Missing	410 839 (5.1)	4361 (0.1)
Status on 30 June 2021		
Active	5 801 280 (72.2)	5 801 280 (100)
Transferred out	1 545 850 (19.2)	–
Death	689 818 (8.6)	–

IQR, interquartile range.

### The Information System for Research in Primary Care

The Information System for Research in Primary Care (SIDIAP; www.sidiap.org) database includes routinely collected data by >30 000 professionals from the ICS. During the 1990s, the ICS created a computerized programme [*estació clínica d’atenció primària* (e-CAP)] for the recording of information during primary care visits in a structured format that has been in use since 2005. In 2010, the ICS and the Institute for Primary Health Care Research Jordi Gol i Gurina (IDIAPJGol) created SIDIAP, which included the data collected through the e-CAP programme since 2006. SIDIAP was designed to provide a valid and reliable database of selected information from the patients’ electronic health records (EHRs) for research.^3^

Table 1 presents the main characteristics of the SIDIAP population. The database has information on 8 036 948 people, of whom 5 801 280 (72.2%) were still active as of 30 June 2021, 1 545 850 (19.2%) had been transferred out of the database (i.e. individuals who had moved out of the catchment area of SIDIAP) and 689 818 (8.6%) had died. Individuals are automatically incorporated into SIDIAP if they are registered in the public health system and have been assigned to a primary care centre of the ICS. The only requirement to do the self-registration in the public health system is to live in Catalonia (based on a census certificate). The registration process is free of charge and can be done online (without having to go to a primary care centre) or in person at a primary care centre. For births that take place in public healthcare facilities, the facility registers the newborn in the public health system. Individuals can subsequently leave the database when they move out of the catchment area (based on the census certificate) of SIDIAP or die. The median follow-up time of the population is 15.2 [interquartile range (IQR): 6.2–15.5] years (Table 1).

**Figure 2:**
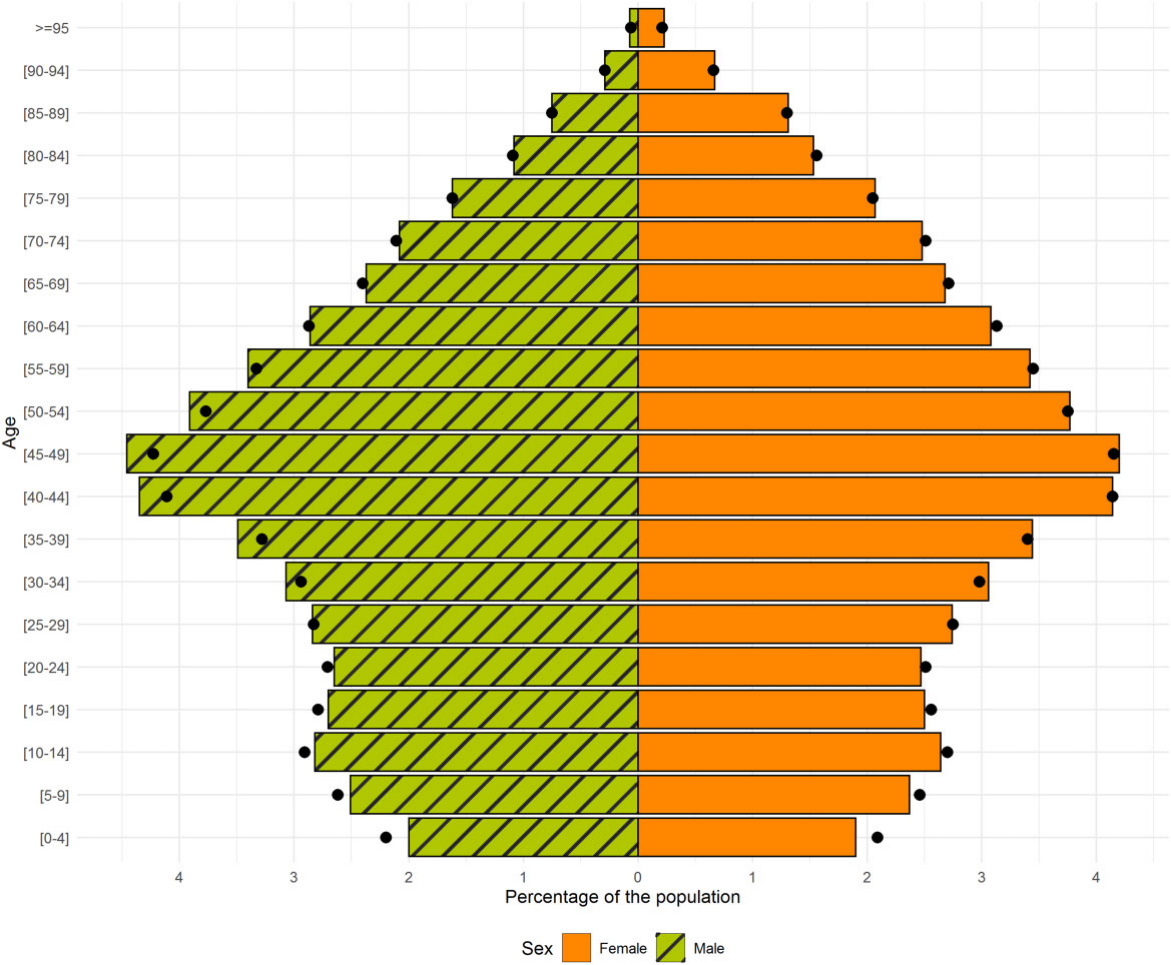
Age and sex distribution in the current Information System for Research in Primary Care population and in the general population of Catalonia The Information System for Research in Primary Care data used for this graph was extracted on 30 June 2021. The data of the population of Catalonia were obtained from the Instituto Nacional de Estadística (National Institute of Statistics) website for the year 2020, ‘Población por comunidades, edad (grupos quinquenales), Españoles/Extranjeros, Sexo y Año’ tab, available from https://www.ine.es/jaxi/Tabla.htm?path=/t20/e245/p08/l0/&file=02002.px&L=0.

The current SIDIAP population (as of 30 June 2021) has a balanced sex distribution (50.7% are female) and a median age of 44 years (IQR: 25–60). The sex and age distribution of the SIDIAP population is similar to that of the general population in Catalonia (Figure 2). The large majority of the SIDIAP population is of Spanish nationality (83.9%), lives in urban areas (88.4%) and resides in the Barcelona region (75.3%) (Table 1). Interestingly, whereas the majority of the SIDIAP population resides in the Barcelona region, as seen in Figure 1, SIDIAP has a population coverage of ≤24% for several Basic Health Areas of Barcelona City.

## Data collected

SIDIAP is a dynamic database containing pseudo-anonymized data recorded in primary care centres (e.g. disease diagnoses, lifestyle information, clinical parameters, etc.) on a daily basis. It also contains external information related to the primary care visit such as pharmacy dispensations and results of laboratory tests, among others (Table 2). Although SIDIAP systematically collects data since 2006, information prior to this date is also available due to professionals recording data retrospectively and to the data transferred from paper to the EHRs in certain centres during the computerization process. The database is updated every 6 months and is structured in data domains, each containing the person’s pseudo-anonymized identifier, which allows linkage between them. Although the number of available data domains grows over time, a description of those most widely used is provided in Table 2.

**Table 2:** Description of key data available in the Information System for Research in Primary Care database

Type of data collected	Data domain in the SIDIAP database	Number of people with at least one entry for the data domain	Total number of entries for the data domain	Key information recorded in the data domain
Socio-demographics	Population	8 036 948	8 036 948	Date of birth, sex, date of entry to the database, date and cause of exit (death, transferral out of the database) if applicable, nationality (grouped into 11 categories)
	Socio-economic variables	8 036 948	8 036 948	Individual income level (<18 000€, between 18 000€ and 100 000€, >100 000€ per year), deprivation indices measured at the census tract level (MEDEA^a^, IP2011^b^) and the basic health area level (ISC)^c^, requested pharmaceutical contribution (exempted, or contribution of 10%, 40%, 50%, 60%), maximum monthly pharmaceutical contribution (without limit, or up to 8.23€, 18.52€ or 61.75€)
	Regional variables	8 036 948	18 036 948	Type of residential area (rural^d^ or urban), province (Barcelona, Tarragona, Girona, Lleida) and basic health area (*n* = 388) where the person resides; productive unit (333 categories, e.g. EAP Vallirana, EAP Salt, EAP La Garriga, etc.), sanitary scope (10 categories, e.g. Barcelona, Girona, Terres del Ebre, etc.).
	Complexity	8 036 948	8 036 948	Clinical risk group (indicator based on the person’s co-morbidities), state of fragility (categorizes persons into ‘with chronic complexity’, ‘with advanced chronic disease’ or none) and whether the person lives in a nursing home (yes, no)
Health conditions	Primary care diagnoses	7 531 524	171 215 580	ICD-10 code, dates of start and end (if there is any), SIDIAP grouper (e.g. ‘Arthrosis’ includes two ICD-10 codes with its descendants) and type of productive unit (hospital, primary care team, mental health centre, etc.) that registers the health problem
	Sick, maternity or paternity leaves	3 191 357	16 651 062	Coding of the health problem causing the sick leave (ICD-10) and dates of start and end of leave
Medications and vaccines	Prescriptions	7 083 845	320 380 795	ATC code, treatment group (e.g. anxiety, hormonal replacement therapy, hypertension), dates of start and end of prescription, posology, frequency of intake and DDD of the medication, speciality of the health professional (e.g. general medicine, gynaecology, paediatrician) and setting (hospital, primary care team, mental health centre, etc.) of prescription
	Dispensations	6 928 471	1 082 492 571	ATC code, dates of start and end of the dispensation, DDD, number of packages dispensed per month
	Adverse reactions	240 047	316 214	ATC code of the drug that produces the adverse reaction and date of occurrence
	Vaccinations	6 443 204	116 615 695	Code, date and dose of administration and grouper of the antigen of the vaccine
Laboratory tests	Analytical variables	5 703 343	1 129 564 883	Biomarker/measurement (e.g. glucose, cholesterol, bilirubin, PCR), date of test/measurement, result value and unit of the test, and interpretation of the value (in four categories: positive, negative, indeterminate, inconclusive or through free text if applicable)
	Serology	2 051 806	11 170 439	Serological test (e.g. hepatitis C antibodies, HIV) date and result (i.e. positive, negative, indeterminate)
Clinical practice and lifestyle information	Clinical and lifestyle variables	6 545 968	513 637 096	Clinical measurement (e.g. systolic/diastolic blood pressure, weight, height) and lifestyle variables (e.g. alcohol consumption, smoking status) date and value of the registry
	Request of complementary explorations/tests	4 314 375	21 741 943	Type (e.g. mammography, colonoscopy) and date of the exploration request
Visits	Visits	7 473 119	695 049 273	Visited service, SIDIAP grouper (e.g. dermatology, nursing, general practice), type of productive unit (hospital, primary care team, mental health centre, etc.) that does the visit, place (online, in person at the health centre or at the residence of the person) where the visit takes place
	Referrals	4 738 748	22 042 118	Health service to which the person is being referred to, date and cause (usually suspicion of an ICD-10 code) of referral
Sexual and reproductive health	Variables of sexual and reproductive health assistance	1 566 025	172 218 876	Variable (e.g. year of menopause, breastfeeding, or result of the last mammography), date and value (if none, the result is reported in another variable containing text in free format) of the registry
	Pregnancy	427 193	649 630	Date of last period and of estimated delivery, type of delivery (e.g. voluntary miscarriage, natural miscarriage, c-section), gestational age (in weeks), miscarriage risk (none, low, normal, moderate, high, very high), number of foetuses, trimestral obstetric ultrasounds [containing information about the mother (type of exploration, location of the placenta, type of amniotic fluid, etc.) and the foetus(es) (sex, cardiac activity, cephalic circumference, etc.)]
Paediatric health (<15 years of age)	Paediatric health	1 592 446	263 953 678	Variables related to birth (e.g. gestational age at delivery, sex, weight, height), screening (e.g. neonatal deafness, cystic fibrosis, congenital hypothyroidism), development (e.g. reaction to external stimuli, object manipulation, sphincter control), nutrition (e.g. gluten intolerance, breastfeeding length, beikost consumption), school (e.g. course, integration, performance), hygiene (e.g. correct fingernail, oral and genital hygiene), leisure and sports (e.g. extracurricular and weekend activities, screen time) and sleeping patterns (e.g. dyssomnia, sleeping schedule, snoring)
Other data sources available through external linkage	Diagnoses and medical procedures at hospital discharge^e^	3 223 390	59 748 073	ICD-10-CM/PCS code, SIDIAP grouper, position of the diagnosis/procedure at the time of admission (used to prioritize the diagnoses/procedures that caused the admission), dates and circumstance (urgent, scheduled) of admission, and dates and circumstance (eight categories, including home, voluntary discharge or death) of discharge. From 2016 onwards, type of anaesthesia used, ICU admission and length of it, date of the first ICU admission
	Hospital medication for outpatient dispensing^e^	516 557	14 563 331	ATC code, date of dispensation, content per box (tablets, syringes, suppositories, etc.), code of the pharmaceutical product, reason for the dispensation, price of the dispensation

a Deprivation index (based on five indicators related to work, education, housing conditions) calculated at the census tract level and available for urban areas.

b Deprivation index available at the residential census tract level based on six indicators of employment and education for urban and rural areas.

c Deprivation index calculated at the basic health area level for the assignment of the budgets of the primary healthcare teams in Catalonia valid for urban and rural areas.

d Rural areas are defined as municipalities with a population density of <100 people per km^2^ and/or a population of <30 000 inhabitants.

e Information recorded in all Catalan public hospitals registered in the minimum basic set of hospital discharge data (CMBD-AH) available through linkage to the Data Analysis Program for Health Research and Innovation (PADRIS) of the Department of Health.

ATC, Anatomical Therapeutic Chemical; DDD, defined daily dose; GP, general practitioner; ICD-10, International Classification of Diseases, 10th Revision (CM: Clinical Modification or PCS: Procedure Coding System); ICU, intensive care unit; IP2011, Deprivation Index 2011; ISC, Composed Socio-economic Index; HIV, human immunodeficiency virus; MEDEA, *Mortalidad en áreas pequeñas Españolas y Desigualdades Socioeconómicas y Ambientales*; PCR, polymerase chain reaction.

SIDIAP includes socio-demographic characteristics of the population such as the date of birth (only month and year can be provided to avoid re-identification), sex, nationality, type of residential area (rural or urban), dates of entry and exit (if applicable) and the status at the moment of the data extraction (active, transferred out of SIDIAP or dead). Socio-economic status is captured through individual and ecological indicators. The individual income level (<18 000€, between 18 000€ and 100 000€, >100 000€ per year) and type of occupation (active, retired) are obtained through the pharmaceutical co-payment information.^4^ Social class based on occupation is also available for those individuals who have taken sick leave at least once since 2014. The *Mortalidad en áreas pequeñas Españolas y Desigualdades Socioeconómicas y Ambientales* (MEDEA) deprivation index measures socio-economic status at the census tract level of both the residence and the primary care centre.^5^ In addition, the *Índice de Privación* of 2011 (IP2011) is available at the residential census tract level and the *Índice de socioeconómico compuesto* (ISC) is calculated at the primary care centre coverage area level.^6^^,^^7^

Health conditions are captured via diagnoses registered by healthcare professionals using the International Classification of Diseases (ICD) codification system (dates of beginning and end of diagnosis given by a GP can be obtained). Currently, the Tenth Revision, Clinical Modification version of the ICD-10 is being used.

The database also contains comprehensive information regarding prescriptions and dispensations of medications. This includes the drugs (dosage and drug units per day) prescribed by ICS healthcare professionals (mostly GPs although specialists can also initiate a prescription for chronic medications that are continued by GPs in the midterm and long term) that are financed by the Spanish National Health System and dispensed in community pharmacies (number of drug packages dispensed per month). For each drug, the corresponding code from the Anatomical Therapeutic Chemical (ATC) Classification System, defined daily dose recommended by the World Health Organization, the strength, the number of units per package and the administration route are available.

Data on therapeutic and requested procedures, physical examination results, routine measurements and laboratory tests are also captured. Therapeutic procedures include vaccinations (e.g. antigen and the number of administered doses) and health counselling information. Requested procedures comprise diagnostic imaging (e.g. echography, radiology, etc.), tests and scales (e.g. cognitive, pain, mental health, etc.) used in primary care, as well as other cardiovascular, digestive and respiratory diagnostic procedures (e.g. spirometry results, etc.). Physical examination results and routine measurements refer to blood pressure, weight, height, body mass index (BMI), measurements related to child growth and >500 other parameters (e.g. heart rate, cardiovascular risk calculator ‘REGICOR’, etc.). Laboratory tests include information such as cell count, serology and biochemistry, among others, that are collected in each laboratory and automatically integrated into the individual’s EHR.

SIDIAP also contains lifestyle information. The most widely used indicators include smoking status (categorized into never, former or current smoker) and alcohol intake risk (categorized into no risk, low risk or high risk). The latter is calculated based on the reported amount and the frequency of consumption of alcoholic drinks (e.g. on a daily basis), the type of alcoholic drink and/or whether the consumption is made in risky situations (e.g. pregnancy). This information is converted into standard units of alcohol ingested on a weekly basis and converted into levels of alcohol consumption.

Data regarding primary care visits are available, including the date of the visit and the type of professional consulted as well as the cause and date of referral to specialists.

The database includes detailed pregnancy information such as dates of last period and of estimated delivery, along with the type of delivery, the circumstance of the end of the pregnancy (e.g. type of delivery, abortion, etc.), gestational age and trimestral obstetric ultrasounds, among others. SIDIAP contains information about paediatric (<15 years of age) health (e.g. nutrition, development, screening tests, etc.), collected under the framework of the *Programa de infancia amb salut* (Childhood and Health Program).^8^ In addition, parent–child linkage is available for children and adolescents born or entering the database after 2006.

SIDIAP continues to incorporate new information into the database when needed (e.g. to answer new research questions or to monitor more closely a specific condition or disease, etc.) and possible. For instance, during the coronavirus disease 2019 (COVID-19) pandemic, SIDIAP incorporated additional information needed to investigate this disease (e.g. polymerase chain reaction test results, administered vaccines, etc.) in a timely fashion.

Free text that has been previously anonymized is available when sufficient detail cannot be obtained from the structured data. Further information to complement the structured data or to validate diagnoses needed for research can also be obtained through surveys sent to health professionals administered by the ICS.

**Figure 3:**
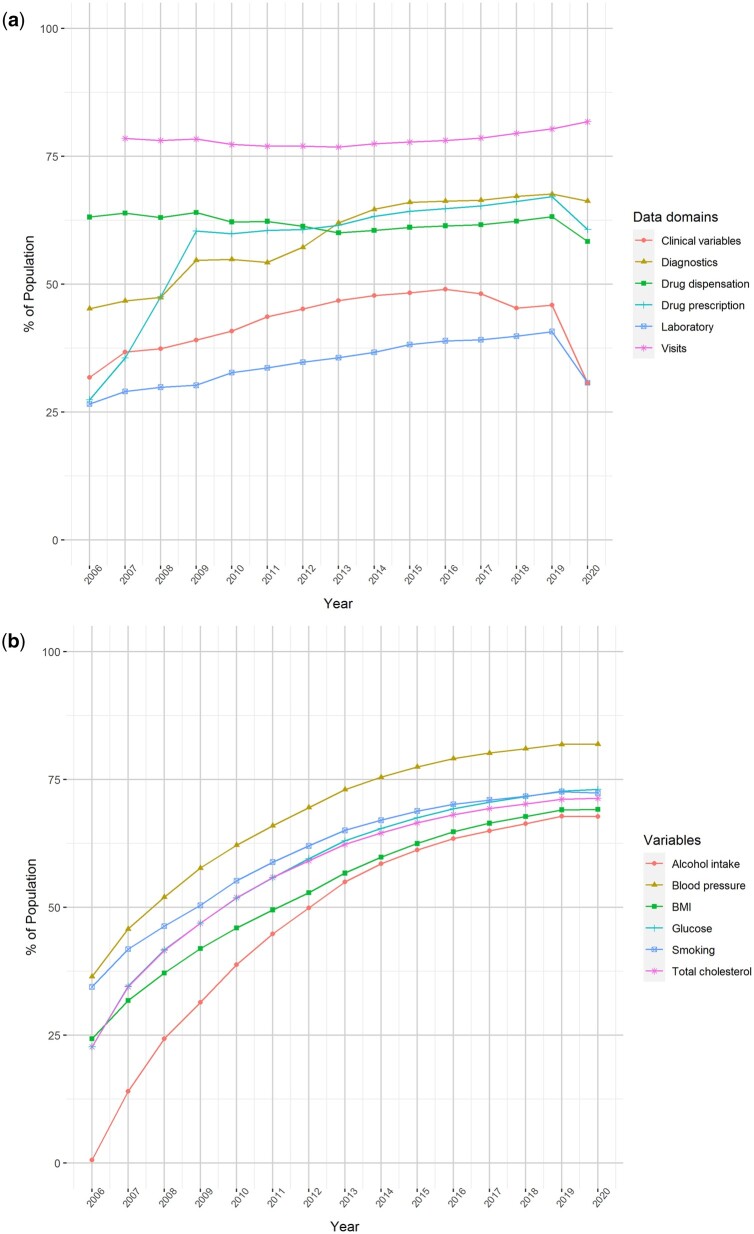
Recording of key variables and data domains in the Information System for Research in Primary Care population by calendar year (a) Proportion of the Information System for Research in Primary Care population with yearly registries of key data domains, by calendar year. (b) Proportion of the population with at least one recording of key variables by calendar year. The data domain ‘visits’ of Figure 3a include all kinds of visits (in person at the primary care centre or at home and telematic). The clinical variables domain shown in Figure 3b contains information about data collected in primary care visits (BMI, blood pressure, smoking status, alcohol intake, etc.). BMI, body mass index.

The growth in the recording of information in SIDIAP over time is shown in Figure 3a and b. For example, in 2019, ∼80% of the SIDIAP population had at least one visit to primary care and >60% had one clinical diagnosis and/or a prescription/dispensation for a medication (Figure 3a). A decrease in the amount of recorded information can be observed in 2020 (likely due to the COVID-19 pandemic). By 2019, 75% of the population had at least one record available of blood pressure and >60% had a record of alcohol intake, BMI, glucose, total cholesterol and/or smoking status (Figure 3b).

### Linkage to other data sources

SIDIAP is a pseudo-anonymized database and does not contain individual personal data. Nevertheless, it can be linked to other data sources on a project-by-project basis through a Trusted Third Party (TTP) using the individuals’ unique personal identifier.

The information recorded in all Catalan public hospitals is registered in the minimum basic set of hospital discharge data (CMBD-AH) and is linked to SIDIAP through the *Programa d'analítica de dades per a la recerca i la innovació en salut* (PADRIS, Data Analysis Program for Health Research and Innovation) of the Catalan Department of Health.^9^^,^^10^ This linkage has been widely used for SIDIAP research and includes the date and cause of hospitalization and discharge, as well as the codes registered during the stay (in ICD-10-CM and ICD-10-PCS, respectively).^11–19^ Data from psychiatric hospitals, outpatient centres of mental health, dispensed medication in hospital settings and emergency rooms can also be obtained through the same linkage process.^10^ In addition, SIDIAP has been previously linked to disease registries of cancer, arthroplasties, dementia, kidney transplants and dialysis, among others.^13–17^^,^^20^^,^^21^ Finally, linkage to urban environment indicators (air pollution, noise, green spaces and built environment) at the census tract level^22^ and to external cohorts at the individual level have also been conducted. An example of the latter is the population-based prospective peripheral arterial disease study (ARTPER) cohort that includes 3786 individuals aged >49 years recruited in 28 primary care centres of Catalonia through random sampling. The participants were given an appointment for an interview, a blood sample extraction and a visit at which anthropometric indicators were measured (including the ankle arm index examination). The collected data were used to estimate the prevalence and associated risk factors of peripheral arterial disease in the general population.^16^

### Data quality

Internal and external validation processes are carried out to determine the data quality of the SIDIAP information at each data update. These include stratifying the data by geographical regions and year in order to identify differences in data collection that need to be harmonized (e.g. recording of a specific information under different codes). The measurement units of variables measuring one characteristic are also homogenized (e.g. transformation of the data from every laboratory that measures haemoglobin to grams per decilitre). Visual inspection of all data included in the database by week is also conducted, allowing one to see temporal patterns in the registry of a certain variable. With this information, the SIDIAP team can issue recommendations to researchers about the most common variable(s) where certain information is recorded (e.g. there are several variables with information concerning the women’s menopausal status and with these visual inspection tools the SIDIAP team can inform the researchers about which related variables have the largest number of records and could be more helpful to capture menopause). Data availability (longitudinality and reliability), plausibility (range checks and unusual values) and consistency are inspected through visualization tools. In addition, before having access to the data for a requested project, research teams have access to a quality-control report. This document contains counts, years, percentiles, maximums and minimums, incidences and prevalences of the data requested for the project, allowing detection of inconsistencies in the data extraction prior to data delivery.

External validation processes of the SIDIAP database mainly include assessing the data recorded in SIDIAP through linkage to external gold standard data sources, by analysing free text or by sending questionnaires to health professionals. The quality of a wide number of data captured in SIDIAP (e.g. cancer, Alzheimer’s disease, dementia, cardiovascular risk factors and musculoskeletal disorders) has been demonstrated in validation studies.^13–16^^,^^20^^,^^21^^,^^23^^,^^24^

## Data resource use

SIDIAP data have been extensively used by national and international institutions to generate real-world evidence. A non-exhaustive list of 223 peer-reviewed published articles and of 306 projects (of which 37 are still ongoing) using the SIDIAP database is available on the SIDIAP website (www.sidiap.org, ‘Projects’ and ‘Dissemination’ tabs). These publications cover a wide range of research topics such as cardiovascular diseases, diabetes, musculoskeletal disorders, respiratory problems, cancer, mental health, multimorbidity, COVID-19, vaccinations; and research areas including pharmacoepidemiology, evaluation of safety and comparative effectiveness research, characterization of a disease, drug utilization, temporal trends of disease, health economics and evaluation of healthcare services, among others.^11^^,^^18^^,^^19^^,^^25–30^

## Strengths and weaknesses

### Strengths

SIDIAP has several strengths. First, the database is representative of the population of Catalonia in terms of age, sex and geographic distribution (Figures 1 and 2). This favours the generalizability of the findings of the studies conducted using SIDIAP to the general population living in Catalonia but also to other comparable regions. Second, due to SIDIAP’s large size, this database can be used to answer research questions that would not be feasible in smaller-sized data sets. Third, the diverse type of data encompassed by this database is also an asset. Not only does SIDIAP include data typically recorded in EHRs (e.g. clinical diagnoses) but also contains socio-demographic information (e.g. socio-economic status or nationality) and lifestyle information (e.g. smoking status or alcohol intake). The parent–child linkage is also a major strength as it allows one to study the impact of parental health and early life exposures on health outcomes during childhood. Furthermore, SIDIAP contains data from external sources such as biomarkers’ information originating from laboratories or prescription and dispensation of drugs, which makes the assessment of drug exposure quite complete. Data from different settings (e.g. disease and hospitalization registries) can also be obtained through diverse linkages, enriching the data available for studies. Finally, SIDIAP is being mapped to different common data models used in European projects. At present, it has already been mapped to the international Observational Medical Outcomes Partnership–Common Data Model (OMOP-CDM), which facilitates and promotes multi-database studies, helps with data management and data analyses, and ensures confidentiality throughout the studies using a federated analysis approach.^31^

### Weaknesses

The SIDIAP database also has weaknesses. Although the database is representative of the population living in Catalonia and regions with similar socio-demographics, it is not necessarily so of other regions of Spain or other countries. In addition, data missingness is a common issue of EHRs (e.g. BMI is not recorded for every participant in the database, as seen in Figure 3b) and a recent measurement of a variable of interest might not be available at the index date for a particular study (e.g. the last BMI measurement available might have been recorded years before the index date). However, methodological approaches such as multiple imputations can be implemented to reduce collider bias in research studies.^32^ Under-reporting of certain variables is also a limitation that can lead to the underestimation of the frequency of a certain exposure or condition (e.g. less severe behavioural or mental disorders might be more likely to go undiagnosed in clinical practice). Furthermore, individual validation of a complete list of events of interest, as conducted ad hoc in cohort or case–control studies, is not possible for large EHR databases and may lead to misclassification. However, algorithms to capture diseases or conditions can be tested in validation studies and allow the quantification of the data quality. Also, relevant information for research might be recorded in unstructured format (i.e. free text) by health professionals. Although advanced techniques to process these data are not yet available in SIDIAP, previously anonymized free text can be manually explored by researchers. Another limitation refers to clinical practice standards and coding that can change over time, giving rise to observed changes in the incidence of a certain condition that might be unrelated to its epidemiology. Finally, due to the primary care nature of this database, studies conducted with SIDIAP could lack the granularity to answer certain research questions. For instance, specialist prescribing, drugs administered in the hospital setting, drugs purchased over the counter and actual drug intake are not available in the database.

## Data resource access

Any researcher is able to request SIDIAP data to conduct a study. A five-step procedure takes place before data access is granted: (i) the researcher(s) must send an application (standardized form available at www.sidiap.org and study protocol) to the SIDIAP team; (ii) the application is approved by SIDIAP’s Scientific Committee which evaluates the scientific quality and feasibility of the proposal; (iii) the study protocol is approved by the Clinical Research Ethics Committee of IDIAPJGol; (iv) the principal investigator of the study must sign a Good Practice form and, in some cases, an agreement between parties is needed; and (v) a meeting between the research team and the SIDIAP team is arranged to discuss the procedures and set the data extraction. Further information is available online (https://www.sidiap.org/index.php/menu-solicitudesen/application-proccedure) or by contacting Anna Moleras (sidiap@idiapjgol.org). Data access is limited to researchers from public organizations and collaboration with private institutions is possible when a study is required by a regulatory agency or for non-commercial studies within a European project financed by the European Commission.

In accordance with current European and national law, the data used in this study are only available for the researchers participating in this study. Thus, we are not allowed to distribute or make publicly available the data to other parties.

## Ethics approval

The use of the data included in the Information System for Research in Primary Care (SIDIAP) is authorized by the Catalan Health Institute (ICS) and Data Analysis Program for Health Research and Innovation (PADRIS) who ensure the pseudo-anonymization of the information. When linkage with other public data sources is required, ICS or PADRIS act as a Trusted Third Party (TTP) to execute the linkage and provide the new data set already pseudo-anonymized; otherwise, informed consent of patients is needed to access their personal data, using the same TTP. SIDIAP does not provide information subject to re-identification and aggregations or deletions are applied in order to protect pseudo-anonymization. The data are managed in a secure server following all the present legal requirements of the General Data Protection Regulation (European Union) 2016/679 and of the Council of 27 April 2016 and Organic Law 3/2018 of 5 December on the protection of personal data and guarantee of Digital Rights.

This study was exempted from the approval of the Clinical Research Ethics Committee of the IDIAPJGol given that the data were directly analysed in the SIDIAP platform and only aggregated results were reported.

## Data Availability

See Data resource access above.
